# Automatic segmentation and measurement methods of living stomata of plants based on the CV model

**DOI:** 10.1186/s13007-019-0453-5

**Published:** 2019-07-03

**Authors:** Kexin Li, Jianping Huang, Wenlong Song, Jingtao Wang, Shuai Lv, Xiuwei Wang

**Affiliations:** 1 School of Mechanical and Electrical Engineering, Northeast Forestry University (NEFU), Harbin, China; 2 School of Forestry, NEFU, Harbin, 150040 China

**Keywords:** Living stomata, Stomata segmentation, Pore measurement, CV model, Image processing, Black poplar

## Abstract

**Background:**

The stomata of plants mainly regulate gas exchange and water dispersion between the interior and external environments of plants and play a major role in the plants’ health. The existing methods of stomata segmentation and measurement are mostly for specialized plants. The purpose of this research is to develop a generic method for the fully automated segmentation and measurement of the living stomata of different plants. The proposed method utilizes level set theory and image processing technology and can outperform the existing stomata segmentation and measurement methods based on threshold and skeleton in terms of its versatility.

**Results:**

The single stomata images of different plants were the input of the method and a level set based on the Chan-Vese model was used for stomatal segmentation. This allowed the morphological features of the stomata to be measured. Contrary to existing methods, the proposed segmentation method does not need any prior information about the stomata and is independent of the plant types. The segmentation results of 692 living stomata of black poplars show that the average measurement accuracies of the major and minor axes, area, eccentricity and opening degree are 95.68%, 95.53%, 93.04%, 99.46% and 94.32%, respectively. A segmentation test on dayflower (*Commelina benghalensis*) stomata data available in the literature was completed. The results show that the proposed method can effectively segment the stomata images (181 stomata) of dayflowers using bright-field microscopy. The fitted slope of the manually and automatically measured aperture is 0.993, and the R^2^ value is 0.9828, which slightly outperforms the segmentation results that are given in the literature.

**Conclusions:**

The proposed automated segmentation and measurement method for living stomata is superior to the existing methods based on the threshold and skeletonization in terms of versatility. The method does not need any prior information about the stomata. It is an unconstrained segmentation method, which can accurately segment and measure the stomata for different types of plants (woody or herbs). The method can automatically discriminate whether the pore region is independent or not and perform pore region extraction. In addition, the segmentation accuracy of the method is positively correlated with the stomata’s opening degree.

## Background

Stomata are structures that are found in the above-ground parts of all terrestrial plants and account for approximately 95% of gas exchange [1]. The stomata are composed of pores formed by a pair of guard cells. Stomata regulate the exchange of water vapour and CO_2_ between the plant and the atmosphere through changes in the aperture of the pores. They play a pivotal role in controlling the balance between the water loss and carbon gain [2–6]. Therefore, research on stomatal behaviour has been a hot topic in the field of botany. To study the behaviour of stomata, one must be able to calculate the morphology of the pores and quantitatively describe the behaviour of the pores.

There are many methods to measure the morphology of the pores. Omasa and Onoe [7] first conducted stomata measurement studies on sunflowers (*Helianthus annuus* L cv. Russian Mammoth) using Fourier transform filtering to remove the scanning lines and an unsharp masking and thresholding method for the pore region extraction. The parameters of the pores (major and minor axes and area) are obtained by translating and rotating the principal components of the extraction region. This method performs the transformation in the frequency domain of the image, and the calculation costs are large. In addition, the threshold adjustment needs to be performed manually according to the different stomata images, and it is not an automatic segmentation method. Sanyal et al. [8] used the watershed method to perform the stomatal segmentation of SEM images, and used their morphology to remove noise and the Sobel operator to extract the stomatal edges. This is an edge-based method that performs poorly when the edges of the pores are discontinuous or there is more noise. Laga et al. [9] used template matching technology to identify wheat stomata, but the calculation of the stomatal morphological parameters is complex, and the template parameters are only valid for wheat stomata. New templates need to be constructed when handling the stomata of other species. Therefore, the algorithm is less versatile. Liu [10] uses the MSERs (maximal stable external regions) to detect ellipses in grape stomatal images and identify stomata, but this method requires manual interaction when selecting the ellipses, which is a semi-automatic method that assumes that the stomata shape is elliptical. Jayakody [11] proposed a method for automatic pore detection and measurement for grapevines. It combined the threshold with the morphological skeleton to extract the stomatal edges and calculate the parameters for ellipse fitting such as the major and minor axis of the pores and the eccentricity. The algorithm assumes that the stomata are close to the centre of the region of interest (ROI) and that the length of the skeleton remnant needs to be specified. If the pore size (different species) changes, the algorithm will fail to identify the pores. Therefore, the algorithm is only applicable to grape species. Toda et al. [12] used facial recognition technology to perform automatic pore measurement of the stomata for dayflowers (*Commelina benghalensis*). This method uses adaptive threshold technology to calculate the pore parameters. However, the algorithm needs to manually define the ranges of the parameters (such as the area, solidity, major axis length, and centroid coordinates). When the sizes or shapes of the stomata change, the algorithm will fail to identify the pores. Therefore, the above methods are only applicable to a specific plant, such as sunflowers, grapes, wheat or dayflowers, and the data collection process requires picking the leaves and tearing off the epidermis to make a micro-slide and acquire the images using bright-field microscopy. Once the pore size and the type or the conditions of image acquisition are substantially changed, the method mentioned above will not successfully identify the pores.

In this paper, we aim to develop a general method for the automatic segmentation and measurement of plant stomata, which is a level set method based on the Chan-Vese (CV) model [13]. Pore region extraction is accomplished by evolving the energy function, and the pores’ morphological parameters are obtained using the ellipse fitting technique. The method does not rely on image gradient information and can overcome the edge leakage and poor anti-noise ability based on the edge information model. It is suitable for segmentation and measurement of any plant stomata and does not require prior information about the stomata. In addition, this is the first time that research has been conducted on the behaviour of living stomata of plants in situ.

The remainder of this paper is organized as follows. In the “Methods” section, detailed steps and examples of stomata segmentation and pore measurement are given. The experimental results of the study and comparisons with existing methods are presented in the “Results” section. The discussion and conclusion are given in the last section.

## Methods

The purpose of this study was to develop a general automatic stomata segmentation and pore measurement method. It requires the input of a single stomata image (manual or automatic detection) and outputs the morphology parameters of the pores. The method comprises 5 steps: (1) detect and crop a single stomata image as the input, (2) convert the image to greyscale, (3) conduct level set segmentation based on the CV model (with the ROI image centre as the initial position), (4) conduct region shape analysis, and (5) conduct post-processing and ellipse fitting.

**Fig. 1:**
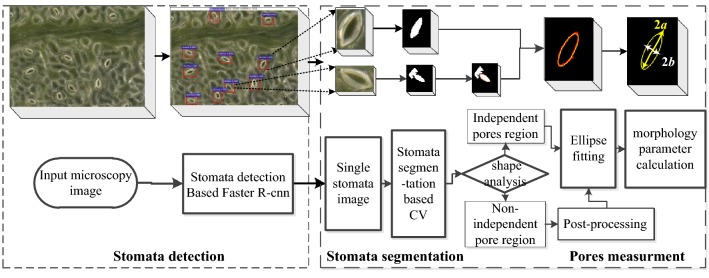
Flow chart of stomata segmentation and measurement. The red contour line is the segmentation contour from the proposed method, and the yellow contour line is the fitted ellipse

The overall flow of the method is shown in Fig. 1. Each 1000× microscope image contains approximately 10–20 stomata, and each 500× magnification image contains approximately 30–40 stomata. First, the stomata are detected using the Faster-RCNN [14], and then the detected stomata are cropped to generate a single stomata image. Second, the single stomata image is used as an input for the stomata segmentation algorithm, and the level set segmentation based on the CV model is implemented (the ROI centre is taken as the initial position).The output is the stomata pore region. Next, shape analysis is performed on the binary image of the pore region to determine whether the segmentation result is an isolated region or not. If it is not an isolated region, morphological post-processing will be executed in order to disconnect the non-isolated regions and obtain an isolated pore region. Conversely, if it is an isolated region, then one can proceed directly to the next step: ellipse fitting. Finally, boundary extraction (red line) and ellipse fitting (yellow line) are performed on the isolated stomata pore region, and the morphological parameters (the major and minor axes, area, opening degree, etc.) of the pore are output. The integral algorithm is automatically operated in series without the prior information assumption for the stomata, and it is independent of the stomata pore dimensions, the opening degree and the data collection conditions. It is a general segmentation method. The details of each step are described below. It should be noted that the acquisition of the clear image of the full field of view is a key preparation step, and the depth composition technique is used in this paper. The image acquisition experiment parameters are detailed in the image acquisition section.

## Stomata detection

### Architecture of the detection model

**Fig. 2:**
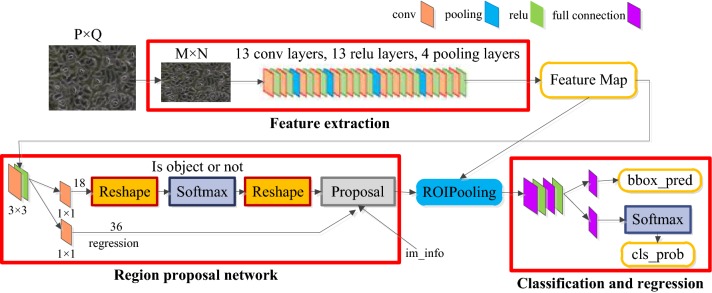
Architecture of the faster R-CNN network

The living stomata were detected using the Faster region-based convolutional neural networks (Faster-RCNN), which is a deep learning method for the detection of objects in natural images [15, 16]. The network architecture is shown in Fig. 2 [14].

The Faster R-CNN network consists of the following four modules. (1) Feature extraction. The Faster R-CNN uses VGG16 to extract feature maps of the input images, and these feature maps will be used for the subsequent region proposal network (RPN) layers and fully connected layers. (2) RPN. The RPN network is used to generate the region proposals. First, several of anchor boxes are generated. Then, softmax is used to determine whether these anchors belong to the foreground or background (whether it is the object or not) after they are cropped and filtered. In addition, another branch named the bounding box regression is used to correct the anchor boxes and form more precise proposals. (3) ROI pooling. The proposals that are generated by the RPN and the feature map that are obtained in the last layer of the VGG16 are combined in order to generate a fixed size proposal feature map, and are prepared for the next step, which is the fully connected operation of target recognition and location. (4) Classification and regression. The fully connected operation of a fixed-size feature map is performed, and then the object classification is completed using softmax. Furthermore, the smooth L_1_ loss is used to complete the bounding box regression operation and get the exact location of the object.

The open source codes [17] of Faster R-CNN detection algorithm is implemented under the TensorFlow framework. The data set consists of 1000 stomata images with 500 images for each of two magnifications (500× and 1000×). The resolution of the images is resized to 800 × 600 in order to train the Faster R-CNN. The ratio of the number of images in the training set, verification set and test set is set to 400/400/200 prior to the model’s training, and the training was performed with the following parameters: the learning rate that was used in the first 50,000 iterations was 0.001, the rate in the last 20,000 iterations was 0.0001, there were 200 epochs, and the batch size was 64. The loss function is

$$L\left( {\left\{ {p_{i} } \right\},\left\{ {t_{i} } \right\}} \right) = \frac{1}{{N_{cls} }}\mathop \sum \limits_{i} L_{cls} (p_{i} ,p_{i}^{*} ) + \lambda \frac{1}{{N_{reg} }}\mathop \sum \limits_{i} p_{i}^{*} L_{reg} \left( {t_{i} ,t_{i}^{*} } \right)$$

where *L*_*cls*_ is the Softmax Loss, *L*_*reg*_ is Smooth *L*_1_ Loss, $\lambda = 10$, and the details of other parameters can be obtained from literature [14].

$${\text{smooth}}_{L1} \left( x \right) = \left\{ {\begin{array}{ll} {0.5x^{2} } &\quad {{\text{if}} \, \left| x \right| < 1}\\ {\left| x \right| - 0.5} &\quad {\text{otherwise}} \\ \end{array} } \right.$$

### Validation of the detection model

1000 images of stomata were used in the datasets, Which include Chinese Necklace Poplar and Black poplar. We use 400 images as the training set, and another 400 images as the verification set and the remained 200 images as the test set.

**Fig. 3:**
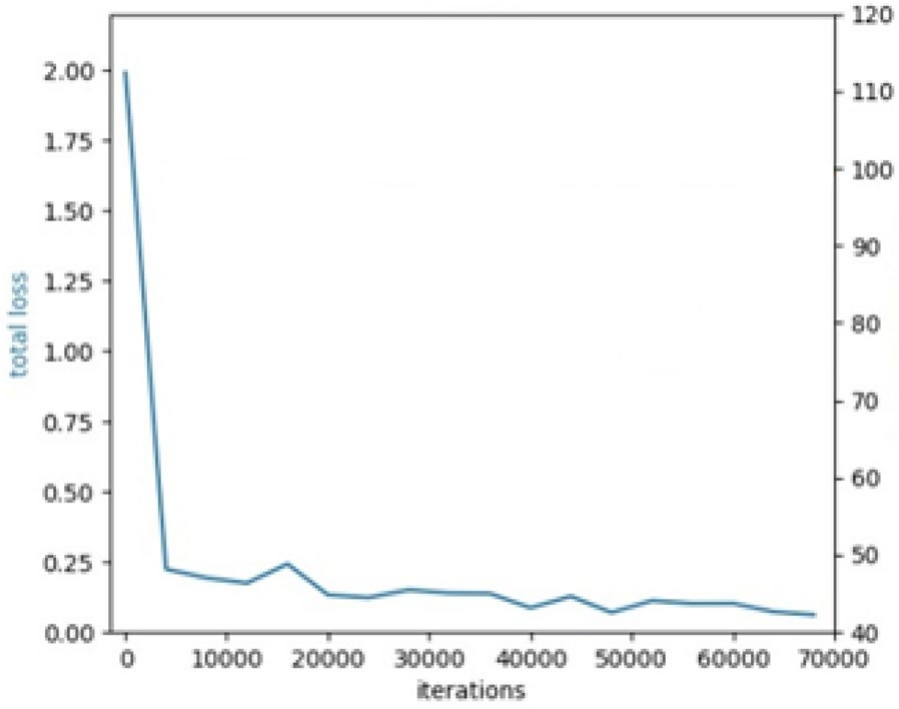
Loss curve of the detection model

The loss curve for training the model is shown in Fig. 3. We detected 1290 stomata from 1316 (Ground truth of the 200 images) stomata using this model successfully, and the recall is 98.02%. The precision is 100%. There is no false detection for the test set.

**Fig. 4:**
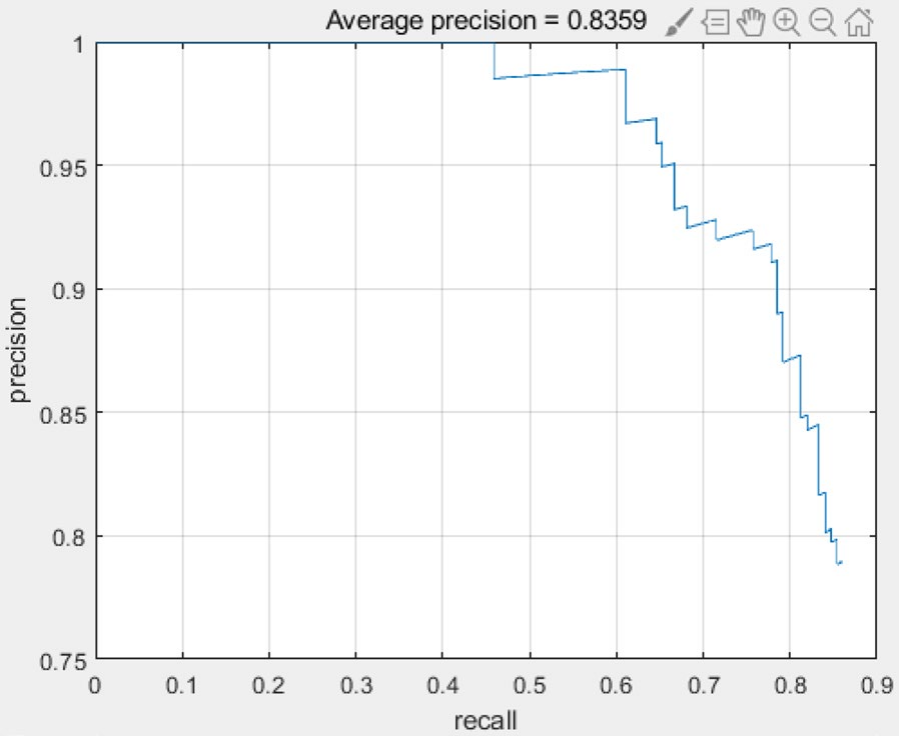
Detection performance of the model for the data of dayflower

**Fig. 5:**
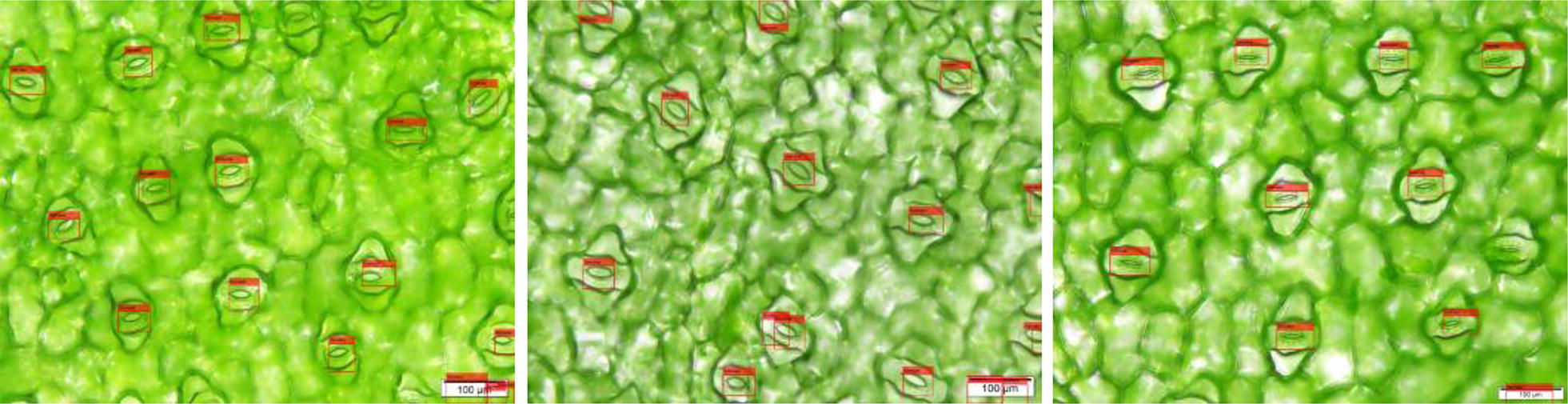
Detection results of the dayflower by the detection model

In order to evaluate the performance of the Faster-RCNN for different data, the data of dayflower provided in the literature [12] was tested using the model. The r-p curve of the model are shown in Fig. 4. The visualization results of the dayflower by the detection model are given in Fig. 5. The recall and the precision is 86.31% and 83.59%, respectively.

### Stomata segmentation

The Chan-Vese model is a geometric active contour segmentation model based on the level set method, which is an improved method of the classical level set [13, 18]. The model uses the average value of the greyscale inside and outside the region as the energy function in order to segment the object region by energy function optimization. Because it has strong adaptability to changes in the topology of the object, it is widely used. It does not rely on image gradient information, can better overcome defects such as edge leakage and poor anti-noise ability based on edge information model, and has global attributes. Perfect segmentation results can also be obtained when the image boundary is discrete or blurred.

The internal and external energy functions of the CV model are defined as follows:

$$E^{CV} \left( {C,C_{1} ,C_{2} } \right) = \mu \cdot Length\left( C \right) + \nu \cdot Area\left( {inside\left( C \right)} \right) + \lambda_{1} \cdot \mathop \int \limits_{outside\left( C \right)}^{{}} \left| {I\left( {x,y} \right) - C_{1} } \right|dxdy + \lambda_{2} \cdot \mathop \int \limits_{inside\left( C \right)}^{{}} \left| {I\left( {x,y} \right) - C_{2} } \right|dxdy$$

where *C*_1_ = average (*I*(*x*, *y*)), outside(*C*); and *C*_2_ = average (*I*(*x*, *y*)), inside (*C*).

The length (*C*) is the length of the closed contour *C*. The area (*C*) is the inner area of *C*. *λ*_1_ and *λ*_2_ are the weight coefficients of the respective energy terms. The first two, *μ* and *ν*, are called “smooth terms”, and they control that the curve maintains a certain smoothness during the evolutionary process. The latter two are called “fitting terms”, and they mainly move the segmentation curve to the edge of the image in order to minimize the fitting error. The position of the final segmentation contour *C* and the unknown constants *C*_1_ and *C*_2_ are obtained by minimizing the energy function.

The steps of the CV model algorithm are as follows.

1. Initialize the level set.
2. Calculate the average grey value of the foreground C_1_ and background C_2_.
3. Each point of the level set is adjusted (evolved), and if the grey value of the current point is close to the foreground estimate, the value of the point level set increases, and vice versa.
4. Once the solution stabilizes, the algorithm stops.

The stomata segmentation algorithm that is proposed assumes that the stomata pores are close to the centre of the ROI, and the centre of the single stoma image is used as the initial point of the evolution of the energy function in the CV model. The whole segmentation process does not need to manually specify the initial position, and does not require prior information about the stomata, which is a fully automatic method for stomata segmentation. The flow chart of the segmentation algorithm is shown in Fig. 2.

A visual representation of the level set segmentation algorithm for living stomata is shown in Fig. 3.

Our aim is to find the stomata pore regions. After segmentation by the CV model, the stomata pore region is obtained and the algorithm outputs the binary image of the stomata pore. Next, the geometric parameters of the pores are calculated.

### Stomata pore measurement

The stomata pore regions that are segmented by the CV model maybe non-independent regions, which have branches or connections to other regions(see Fig. 5b). Therefore, a morphology post-processing technique is used to disconnect the non-independent regions before the pore measurement, and the algorithm must automatically determine whether the segmented region is independent or non-independent.

The stomata pore measurement algorithm is composed of region shape analysis, morphological post-processing, region filling, boundary extraction, ellipse fitting, and parameter calculations. The outputs are the length of the major and minor axes, the opening degree and the pore area. The flow chart is shown in Fig. 4.

The region shape analysis is a key step for this algorithm. It can determine whether the segmented region is independent or not. If it is a non-independent region, a post-processing step is added to disconnect the connection. In this paper, morphology erosion and dilation are used to disconnect the non-isolated regions. The Solidity attribute is used to automatically determine whether the segmented region is an independent region or not. (For a non-independent region, Solidity < 0.85, which represents the concavity and convexity of the region.) A visual representation of this algorithm is shown in Fig. 5.

The Solidity is calculated as follows:

$${\text{Solidity}} = {\text{Area}}/{\text{Convex}}\,{\text{Area}}$$

where Area is the area of region, and Convex Area is the area of the smallest convex polygon.

It indicates the degree of Solidity (concavity and convexity) of the region.

**Fig. 6:**
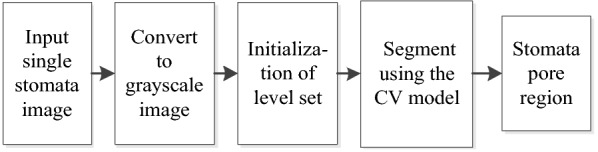
Flowchart of the pore region segmentation for living stomata

For most stomata images in our data, the stomata regions that are segmented by the CV model are independent and do not need the post-processing step, as shown in Fig. 6. In this situation, the Solidity of the region is greater than 0.85, and the ellipse fitting and parameter calculations can be directly accomplished for the segmented stomata pore region.

## Results

### Data acquisition

**Fig. 7:**
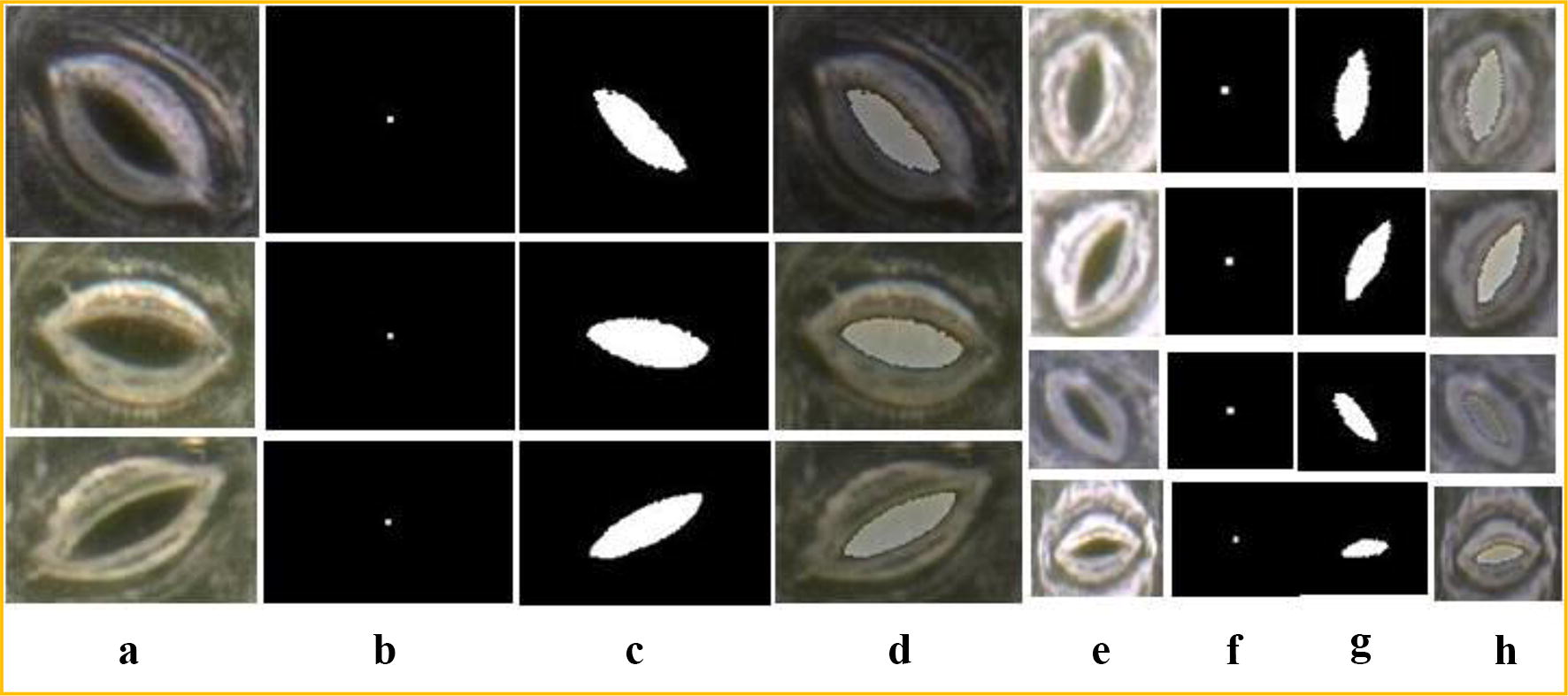
Examples of stomata pore regions that are segmented by the CV model. **a**, **e** Original images (1000× and 500× , respectively). **b**, **f** Initial contour locations. **c**, **g** Segmented stomata pore regions. **d**, **h** Overlays of the segmented pores on the original image

In the experiment, a large depth-of-field microscope observation system, a VHX-2000 [19], from the Keyence Corporation, was used to capture the fully focused images of the living stomata of poplar leaves. The species are one-year-old black poplars (*Populus simonii* × *P. nigra*) and Chinese white poplars (*Populus lasiocarpa* Oliv*)*. Using the depth composition technology of the VHX-2000 system, the images of stomata in the full field of view are taken. The depth composition parameters are set as follows. The depth-of-field interval is 2–4 μm and the number of images included is 20–25 frames, which depend on the flatness of the leaves. The resolution of the captured images is 1600 × 1200 pixels, and we have 4.8 pixels/μm(1000×). A total of 51 microscope images were collected at two magnifications (1000× and 500×), and the total number of stomata is 708. The stoma image acquisition is shown in Fig. 7. Figure 7b is the fully focused microscope images for the living stomata using dark-field microscopy. In addition, a leaf clamp must be used to fix the plant leaves.

### Pores measurement

To evaluate the segmentation performance of the method, the stomata pores were manually segmented by ImageJ under the supervision of botany experts, and the ellipse fitting and corresponding parameters of the pores were measured by ImageJ as the Ground-Truth. The results of this annotation were compared with the segmentation and measurement results of our method.

**Fig. 8:**
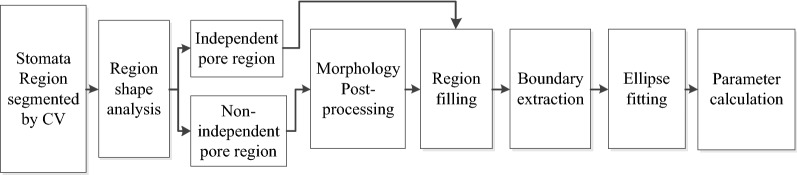
Flow chart of the stomata pore measurement algorithm

Ten of the 708 stomata failed to be identified in the test, and thus, the experimental results show that our method can be used to segment and measure the stomata pores at 1000× and 500× magnifications with high accuracy. The segmentation results of our method are shown in Fig. 8.

According to the geometric knowledge, the semi-major axis and semi-minor axis of a and b for the fitted ellipses are first obtained, and then other parameters are deduced using these two parameters. The calculation formulas are as follows: Area: $A = \pi ab$. Eccentricity: $e = \sqrt {1 - \left( {b/a} \right)^{2} }$. Pore opening degree: $Od = b/a$.

The measurement errors of the major and minor axes of the stomata pores, the area, and the opening degree are obtained. The results are listed in Table 1.

**Table 1:** Morphology parameter calculation results for stomata pores

Number of stomata	Avg. major axis length accuracy	Avg.minor axis length accuracy	Avg. area accuracy	Avg. eccentricity accuracy	Avg. open degree accuracy
698	95.68%	95.53%	93.04%	99.46%	94.32%

In addition, this paper analyses the relationship between the major and minor axes measurement errors and the pore opening degree, as shown in Table 2. The total error is equal to the square root of the major and minor axes errors.

**Table 2:** Relationship between stomata opening degree and measurement error

Opening degree (%)	> 40	(30,40]	(20,30]	(10,20]
Number of stomata	51	287	282	72
Major axis error (%)	3.44	4.32	5.01	6.45
Minor axis error (%)	2.87	3.37	3.91	5.86
Total error (%)	4.83	6.01	6.91	9.51

It can be seen from Table 2 that the segmentation accuracy of the method is positively correlated with the stomata’s opening degree. The greater the stomata opening degree is, the higher the accuracy of the method for measuring the lengths of the major and minor axes. The opening dimension of the stomata has some influence on the algorithm’s performance. The larger the opening degree is, the higher the measurement accuracy of the method.

**Fig. 9:**
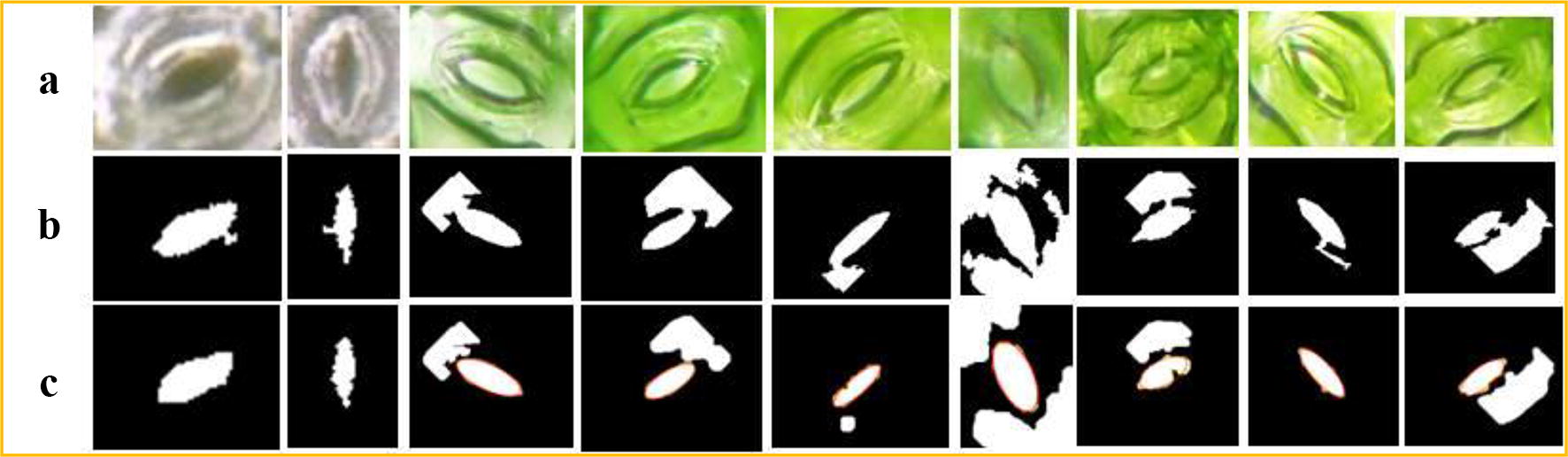
Identification and disconnection of the non-independent stomata pore regions. **a** Original stomata images. **b** Non-independent stomata pore regions that are segmented by CV model. **c** Independent Stomata pore regions

We also give the experimental results that characterize the consistency of the algorithm. The relationship between the minor axis length’s measurement accuracy of our method and that of the manual measurement method is shown in Fig. 9 (for 698 stomata). The regression line’s slope is 1.015 and the R^2^ is 0.9848; therefore, the algorithm has very good consistency.

### Algorithm comparison

**Fig. 10:**
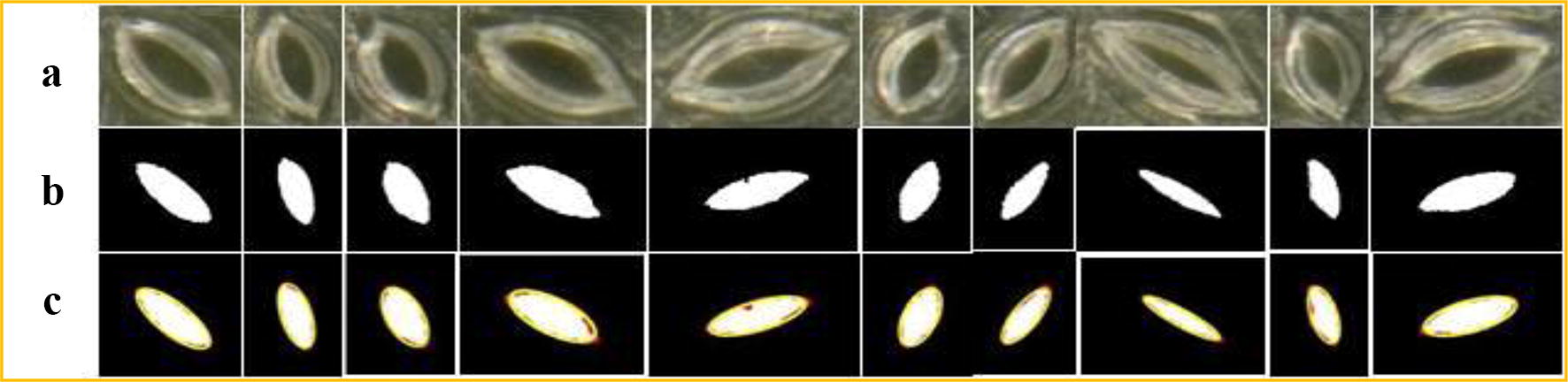
Examples of stomata boundary ellipse fitting. **a** Original stomata images. **b** Stomata regions segmented using CV model. **c** Pore boundary ellipses (yellow lines)

We refer to the stomata as the foreground and the non-stomatal area as the background of the images. The microscope images of the living stomata are feature-rich and include the epidermis, veins, trichomes, stains, etc. Therefore, the stomata that are segmented by the threshold method are not often independent regions and are connected with other areas. In severe cases, even with post-processing techniques such as skeleton extraction and morphological techniques, it is difficult to isolate the stomata regions. The adaptive threshold method in the literature [12] and skeleton extraction combined with ellipse fitting [11] cannot segment the living stomata in the complex image background, especially for low contrast images (such as the living stomata data in this paper), as shown in b and c of Fig. 10. Although the threshold method is simple, it requires good-quality images with clear backgrounds. Often, a complicated post-processing step is required in order to extract the pore regions, and the prior information of the stomata is needed (such as the area range and the stomata perimeter range). Our method based on the CV model proposed in this paper can successfully segment the stomata pores in the complex background, as shown in d of Fig. 10.

**Fig. 11:**
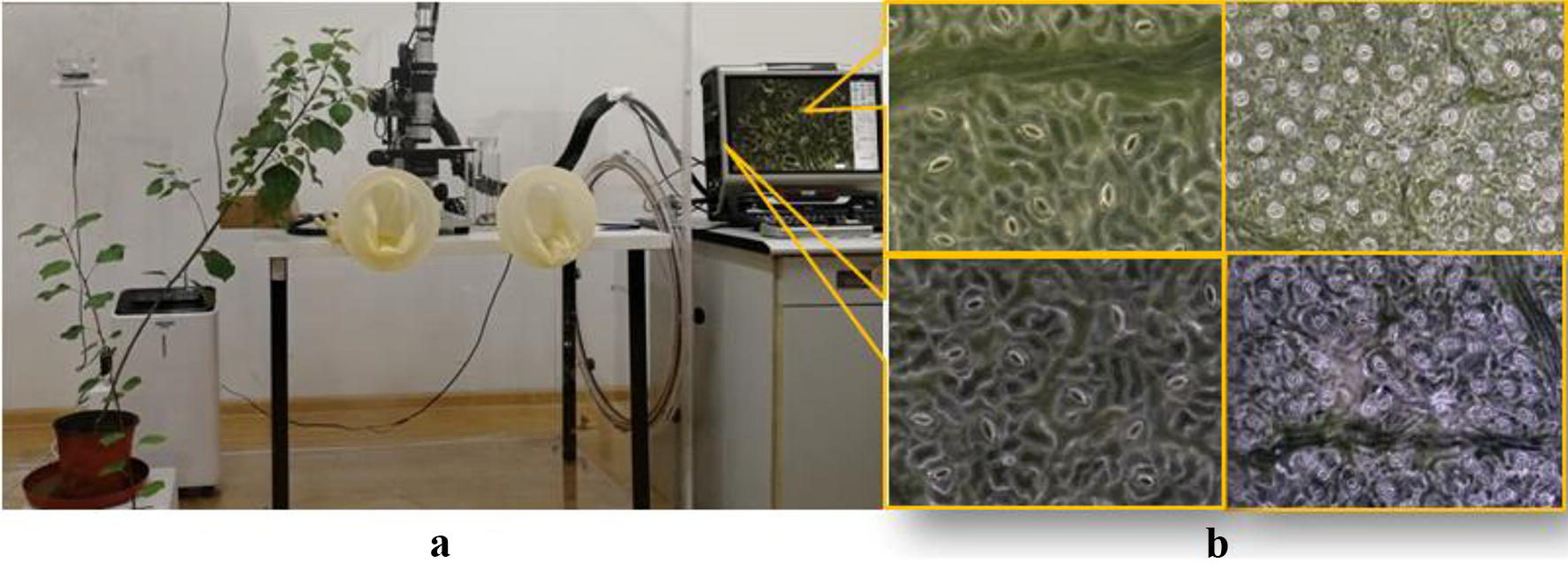
Collection of the living stomata microscope images for black poplars. **a** Image acquisition experiment. **b** Fully-focused images generated by using the depth composition technique

The proposed method can also achieve good segmentation accuracy of the stomata with clear backgrounds (data publicly available in literature [12]). In this paper, the stomata microscope image data that are disclosed in the literature [12] are tested using our method. Due to the significant non-uniform illumination in the bright field environment, the stomata segmentation error is large if the original image is directly segmented using the level set method. Therefore, non-uniform illumination correction is necessary before the segmentation step. Since a reflection region in an image is an area with a higher pixel value, the areas with larger pixel values are replaced with some smaller values to remove the reflections. In this paper, the average grey value of the image is used to replace the pixel value of the reflective area in order to eliminate the reflection, which is followed by the CV model segmentation, and then the stomata pore region can be obtained. The segmentation results with the reflection removal algorithm are shown in Fig. 11.

In Fig. 11, c is the segmentation result by the CV model without non-uniform illumination correction. It can be seen from c that there is a notch in the segmentation results and that the segmentation error is large. Therefore, it is necessary to remove the non-uniform illumination before the pores segmentation step. d is the greyscale image of the stomata after the reflection is removed. When the CV model is run on the images in d, the stomata pore regions that are segmented are relatively intact, as shown in e. The last row f compares the results of the stomata segmentation by our method and the manual method. The red lines are the results of our method’s segmentation, and the yellow lines are the manual segmentation results.

**Fig. 12:**
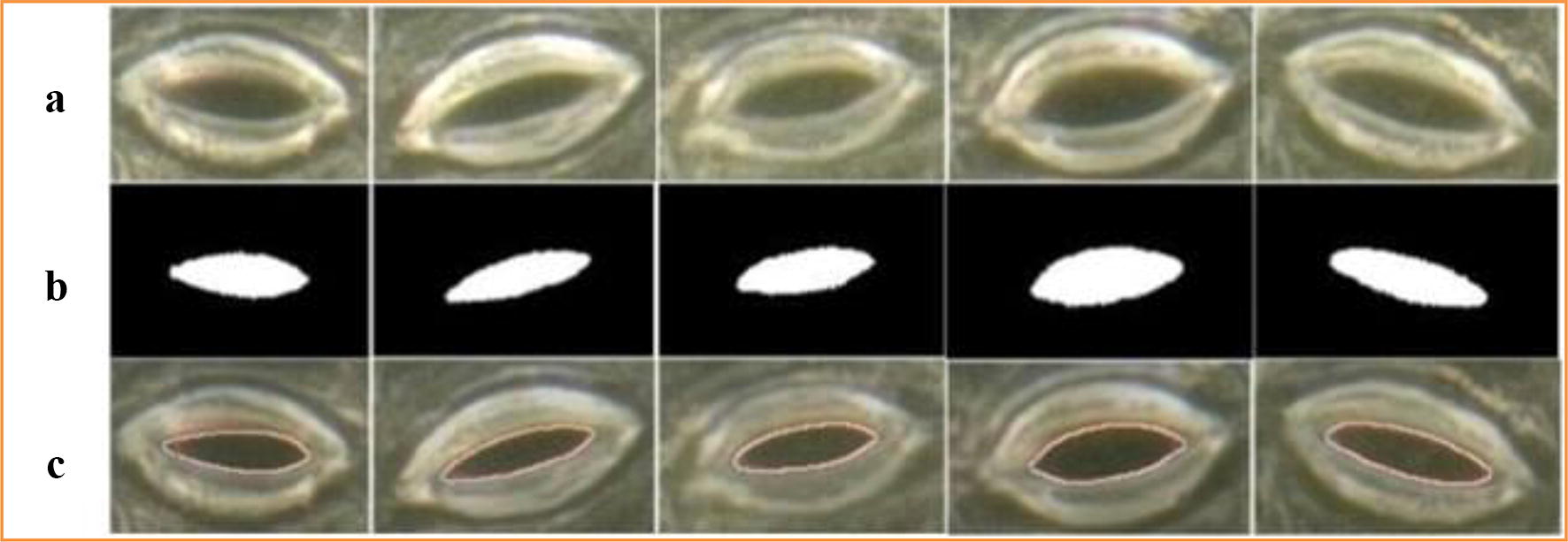
The segmentation results of our method. **a** Original images. **b** Stomata pores that are segmented using proposed method. **c** The contour of b overlays on the original images. (The red line represents the segmentation using our method, and yellow line represents manual segmentation)

To verify the algorithm’s segmentation performance, our method was tested using 188 stomata of dayflower from 16 microscope images from the literature [12]. Among these stomata, 7 stomata failed to be segmented, and 181 stomata were successfully segmented by our method. The fitting relationship between the aperture value (minor axis length) and the manual measurement results of the 181 stomata apertures is shown in Fig. 12.

It can be seen from Fig. 11 that the fitting slope of our method and the manual segmentation is 0.993, and the R^2^ value is 0.9828, which slightly outperforms the segmentation results of literature [12] (slope = 1.0485 and R^2^ = 0.98215). More importantly, the CV-based segmentation method that is proposed in this paper does not require a priori information about the stomata, such as the ranges of the stomata pore area, the major axis length and the pore perimeter. It is an unconstrained, fully automatic stomata segmentation and measurement method. The method has good versatility and is not limited to a specific plant. It is able to segment the stomata of any plant, including non-elliptical shaped stomata.

## Discussion

It can be seen from the experimental results that our method can obtain high segmentation accuracy for most of the stomata images.

Non-uniform illumination correction is not needed for the living stomata in the dark-field microscope images collected in this paper. However, it is necessary for the bright-field images. The reason may be that reflections often result from the stomata pores in the bright-field images, but there are fewer reflections that are generated from the pores in the dark-field images.

**Fig. 13:**
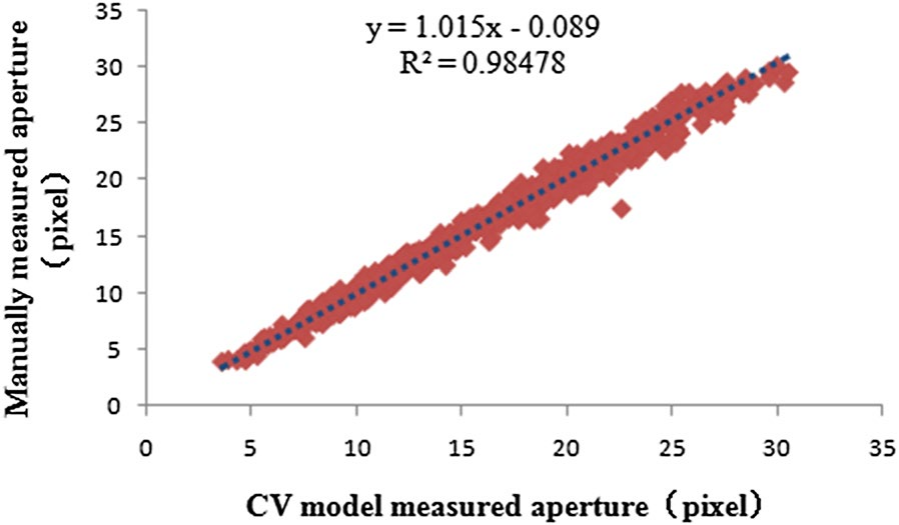
Scatter plot of the automatically quantified stomatal apertures versus the manually quantified apertures (698 living stomata)

The stomata that are discarded in our method are shown in Fig. 13. These include blurred stomata, stomata with too small of opening degrees, and obscured stomata.

**Fig. 14:**
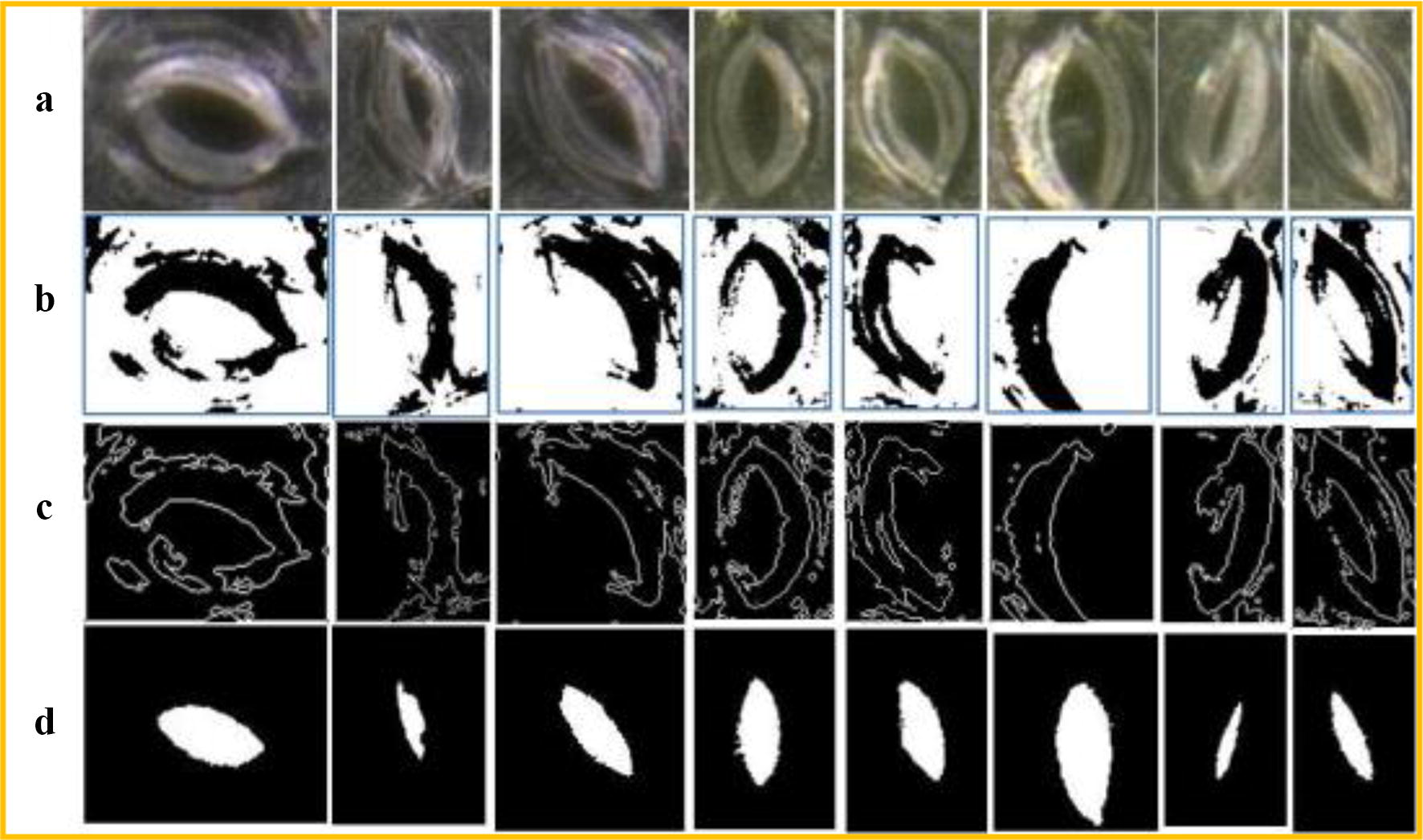
Comparison of adaptive threshold + skeleton extraction and our method. **a** Original stomata images. **b** Segmented results by the threshold method. **c** Segmented results using the skeleton method. **d** Segmented results using the CV model

**Fig. 15:**
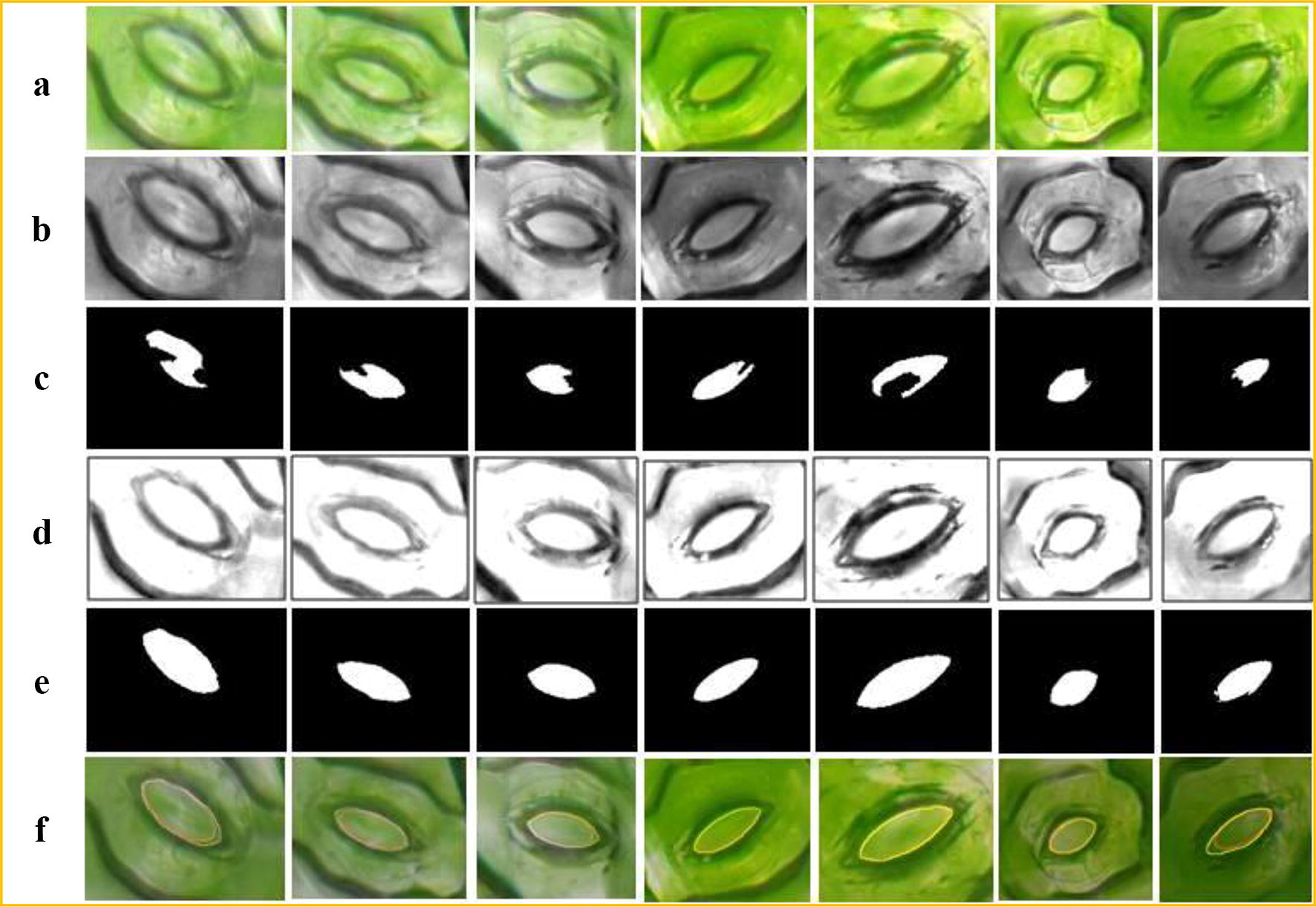
Influence of non-uniform illumination on the results of stomata pore segmentation. **a** Original stomata images.**b** Greyscale images. **c** Segmented results by the CV model without reflection removal. **d** Reflection removal images. **e** Segmented results after reflection removal. **f** Overlays on the original images

**Fig. 16:**
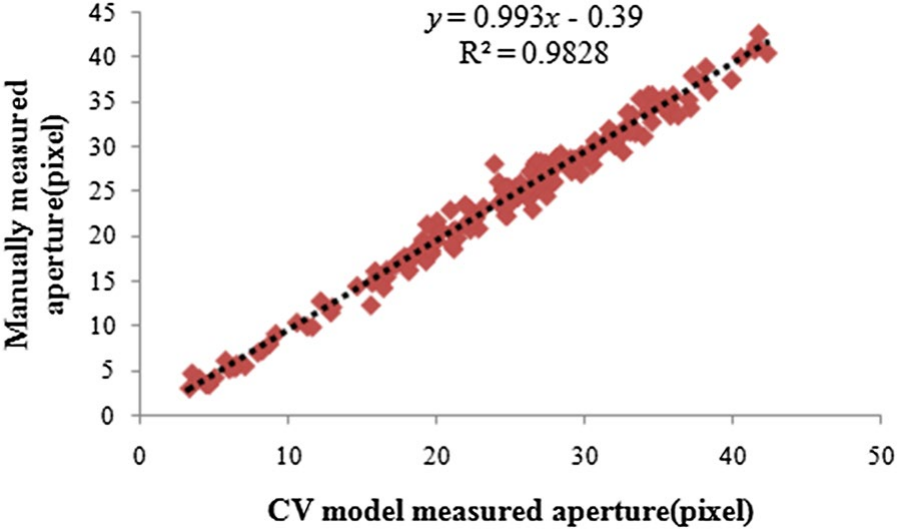
Comparison of the segmentation results using the CV model and manual segmentation (181 stomata Data from literature [12])

**Fig. 17:**
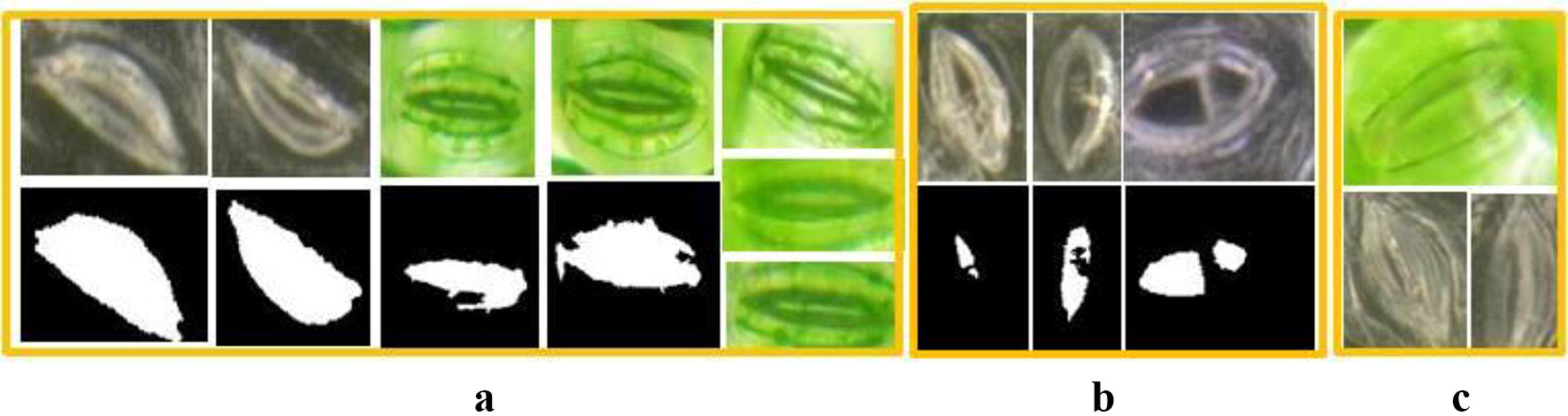
Stomata samples of the images segmentation failures by our method. **a** Stomata with small opening degrees. **b** Obscured stomata. **c** Blurred stomata

Figure 13a shows the stomata images collected under bright and dark fields. The guard cells are also regarded as stomata pore regions, which causes the stomata to be over-segmented. The main reason for the stomata over-segmentation is that the stomata opening degree is too small, and the initial position of the CV does not fall on the pore. Therefore, segmentation results in a stomata apparatus together with the guard cells. b is the three obscured stomata in the dark lighting field. The stomata regions obtained by our method are discontinuous. Post-processing is required to obtain the complete pore region. c is the blurred stomata case. The algorithm will fail to segment these images (Figs. 14, 15, 16, 17).

In addition, the method in this paper only assumes that the pores are located near the centre of the ROI. This assumption is normally true for a detection algorithm. However, for pores with a very small opening (minor axis length < 0.83 μm and approximately 4 pixels for our living stomata images), the initial position of the evolution of the CV model (the centre of the image) usually falls outside the pores, and the segmentation result is over-segmented. Therefore, the stomata with small degrees of opening and deviations from the centre of the ROI cannot be effectively segmented by our method. Fortunately, when the stomata pore opening is too small, the CO_2_ gas exchange is negligible for the stomata, and the stomata can be considered closed.

The proposed method is a region-based level set method, which finds the boundary of the segmentation by calculating the maximum difference between the average grey values inside and outside the region. It does not depend on the gradient information of the image, overcomes the edge leakage of the gradient method and is susceptible to noise. It is a perfect region-based level set segmentation method. However, since the CV-based segmentation method is an iterative optimization algorithm, the algorithm has a long running time, and the average time cost for each stomata segmentation and measurement is approximately 1.18 s (Windows7 environment, Matlab2018a, 3.0 GHz CPU, 4.0 GB of RAM, and 300 iterations).

## Conclusions

In this paper, a general method for the automatic segmentation and measurement of plants’ living stomata based on the CV model is proposed. The method consists of five parts: reflective removal pre-processing(if necessary), CV model-based segmentation, non-independent pore region discrimination, morphological post-processing and ellipse fitting. In this paper, the measurement accuracy of the major-axis length, minor-axis length, area, eccentricity and opening degree of the living stomata were 95.68%, 95.53%, 93.04%, 99.46%, and 94.32%, respectively, after segmenting and measuring the 698 stomata of poplar leaves. The versatility of the algorithm is better than those of existing methods of stomata segmentation. In addition, the algorithm’s consistency is very good for both the bright-field and dark-field stomata images of different datasets, and the R^2^ is greater than 0.98.

This research will be extended to test living stomata of other plant species in the future.

## Data Availability

The datasets and the source code that are used and/or analysed during the current study are available from the corresponding author on reasonable request.

## Abbreviations

CV: Chan-Vese
ROI: region of interest
